# Rethinking Pulmonary Function Tests in Patients with Neuromuscular Disease: The Potential Role of Electrical Impedance Tomography

**DOI:** 10.3390/jcm14238486

**Published:** 2025-11-29

**Authors:** Andrea Vianello, Gabriella Guarnieri, Federico Lionello

**Affiliations:** 1 Department of Cardiac Thoracic Vascular Sciences and Public Health, University of Padova, 35122 Padova, Italy; gabriella.guarnieri@unipd.it; 2 UOC Fisiopatologia Respiratoria, Ospedale-Università di Padova, Via Giustiniani, 2, 35128 Padova, Italy; federico.lionello@aopd.veneto.it

**Keywords:** neuromuscular disease, respiratory failure, electrical impedance tomography, pulmonary function test, assisted coughing technique

## Abstract

An assessment of pulmonary function provides information that can contribute to establishing the severity of respiratory impairment, to predicting the onset of respiratory symptoms, and ultimately to optimizing the care of Neuromuscular Disease (NMD) patients. It is nevertheless well known that conventional Pulmonary Function Tests (PFTs) have several technical limitations and that their accuracy depends to some extent on the patient’s ability to cooperate. For this reason, it is essential to move beyond traditional pulmonary function evaluation in individuals with NMD. A relatively new technology, electrical impedance tomography (EIT) is an easy-to-use, radiation-free imaging technique that may overcome many of the limitations of conventional PFTs by producing real-time images of regional ventilation and tidal volume distribution. As it is safe and independent from patient cooperation, EIT is expected to improve the diagnosis of respiratory compromise and facilitate the implementation of timely, personalized treatments for NMD patients. There are nevertheless technical problems that need to be addressed to facilitate its diffusion in clinical practice.

## 1. Introduction

Insidious, progressive respiratory failure with CO_2_ retention (“ventilatory failure”), frequently worsened by chronic aspiration, secretion retention, and/or pneumonia, is the major cause of morbidity and mortality in patients with Neuromuscular Disease (NMD) [1]. A comprehensive assessment of a patient’s pulmonary function enables clinicians to evaluate the extent of respiratory compromise, to predict the onset of pulmonary complications, and ultimately to optimize respiratory care [2].

Although conventional Pulmonary Function Tests (PFTs) assessing airway pressure, air flow rates, and lung volumes play an essential role in diagnosing, monitoring, and managing the respiratory problems of NMD patients, they nonetheless can be physically demanding and their accuracy depends on the patient’s ability to cooperate. Moreover, while they can evaluate the overall function of the patient’s combined lungs, they are unable to investigate the lungs separately or to evaluate abnormal patterns of lung ventilation distribution [3]. For this reason, it is essential to move beyond traditional pulmonary function evaluation in individuals with NMD. A relatively new technology, electrical impedance tomography (EIT) is an easy-to-use, radiation-free imaging technique that may overcome many of the limitations of conventional PFTs and has the potential to improve the diagnosis and treatment of respiratory compromise in NMD patients.

## 2. Electrical Impedance Tomography: Potential Benefits and Barriers to Its Use in Clinical Practice

**Figure 1:**
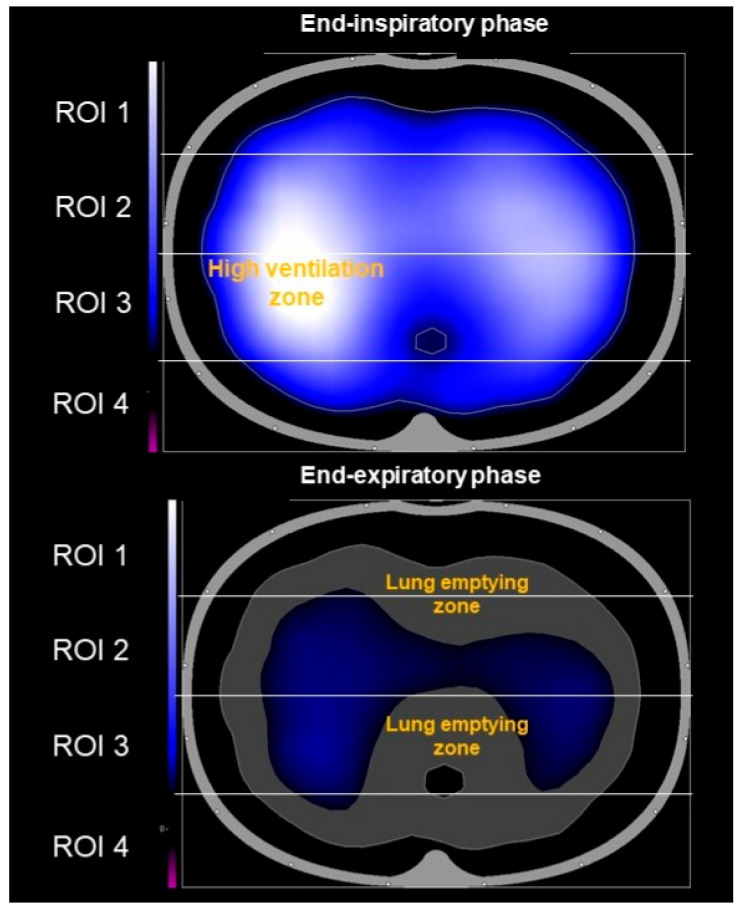
Images obtained via electrical impedance tomography in a healthy subject, displaying differences in tidal volume distribution between the inspiratory and expiratory phase. The blue–white gradient indicates the zone of small and/or large ventilation change, respectively. Differences between volume distribution during the inspiratory and expiratory phases are shown in gray color.

In accordance with the principle that changes in regional air content and/or blood flow modify the electrical impedance of lung tissue, EIT generates images of the regional ventilation and tidal volume distribution of the lungs by measuring the electrical potentials at the chest wall surface using skin electrodes (Figure 1) (Supplementary Materials).

**Figure 2:**
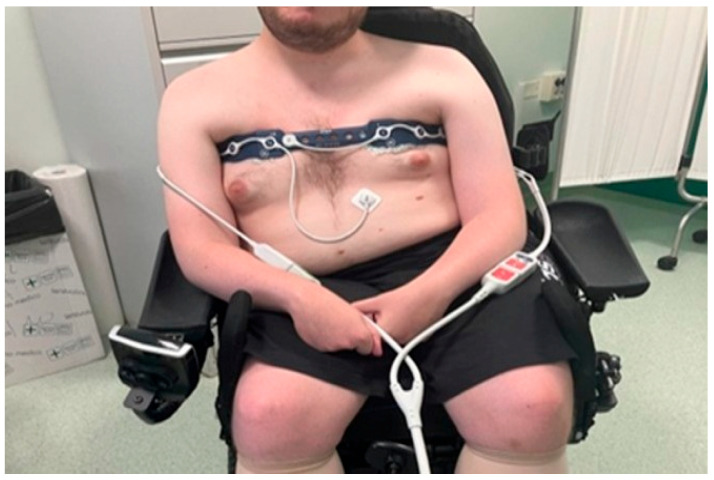
An electrode belt on a young patient with Duchenne Muscular Dystrophy.

Although EIT devices can use 8, 16, or 32 electrodes positioned between the fourth and sixth intercostal spaces of the thorax, in practice, 16 is the most common configuration (Figure 2).

**Figure 3:**
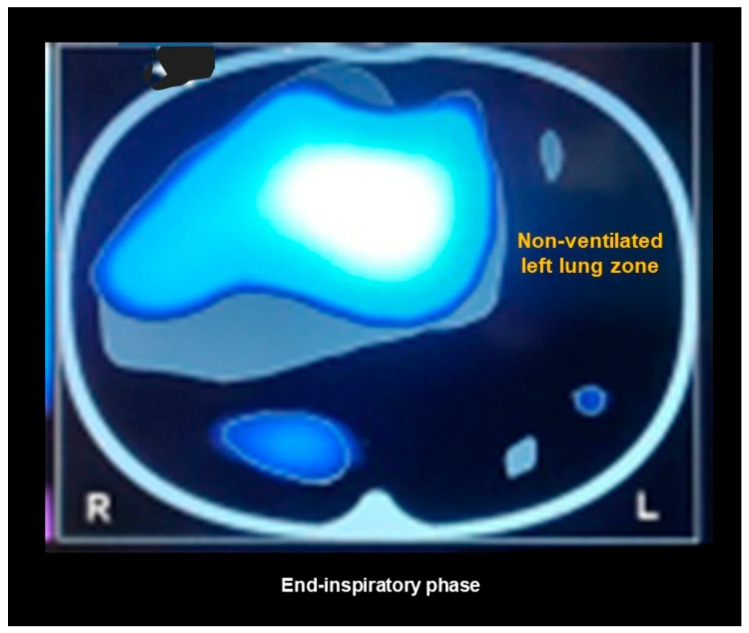
An image obtained via electrical impedance tomography in a Neuromuscular Disease patient with left upper lobe atelectasis. The black color indicates a lack of lung ventilation.

EITs has potential benefits compared to conventional technologies used for pulmonary function evaluation, which can be summarized as follows:

- As opposed to other imaging techniques such CT scans, EIT is a non-invasive, radiation-free, safe tool.
- EIT does not depend upon the patient’s cooperation. Patients do not need to keep their lips sealed around a mouthpiece connected to a spirometer during testing, an enterprise that may be challenging for some NMD patients.
- EIT can be utilized in both the upright and supine position: this is important in the context of patients who may present with very severe orthopnea.
- While conventional PFTs can be fatiguing and non-repeatable due to the maximal effort required to perform the test properly, EIT can be repeated without discomfort or side effects.
- As EIT devices are portable they can be used at a patient’s bedside, a feature that is especially important during an acute phase of a respiratory disease.
- Unlike static anatomical images from CT scans, EIT shows how each individual lung functions in real time by visualizing the distribution of regional lung ventilation and air content.
- EIT facilitates early diagnosis of structural lung abnormalities and can detect atelectasis, pneumothorax, and pleural effusion (Figure 3).

- Finally, EIT is able to detect even small structural changes over short time periods.

The reliability of EIT has been confirmed by comparing its results with those of established conventional imaging techniques, such as computed tomography (CT), photon emission computed tomography and pneumotacography [4]. The safety of the technique has been verified by its inherent non-invasive radiation-free nature and has been confirmed by recent research in adult and pediatric patients, including neonates. Efforts are presently being made to minimize the minor side effects that have been linked to its use, such as skin irritation and allergic reactions [5]. There are only a few contraindications for EIT: for example, electronic devices such as active pacemakers and/or implantable cardioverter-defibrillators as the alternating current can interfere with their function. Other contraindications that have been reported are skin damage or excessive body hair, which may affect electrode contact; severe rib or spinal fractures, which could lead to pain or further damage while the thoracic belt is being placed around the patient’s chest; and extreme obesity, as it may reduce signal quality, making EIT measurements less reliable [6].

Despite significant benefits described above, there are still barriers to EIT utilization in clinical practice, which include the following: the cost of the equipment; a lack of skills and training; the need for at least two skilled operators for performing the examination; poor measurement accuracy in the presence of drains, medications, or thoracic trauma; and data being especially difficult to interpret without dedicated software. As a result, although EIT is no longer a new technology, its clinical use is still at a primary stage. To date, the technique is increasingly being utilized as a diagnostic tool in many Intensive Care Units (ICUs), where the need for real-time insight into a subject’s ventilation distribution is critical for adjusting ventilator settings or changing the posture of an Acute Respiratory Distress Syndrome (ARDS) patient [7]. EIT might also be potentially suitable for monitoring the natural history of lung diseases and the long-term effects of therapy [8]. The preliminary results of studies examining the routine use of EIT for the bedside diagnosis of pneumothorax [9] and, more recently, of pulmonary embolism show that it can reliably detect both [10]. Moreover, EIT’s role in the pre-surgical evaluation of single-lung transplant patients has proven to be crucial [11]; it has also proven to be effective in assessing respiratory mechanics in obese patients [12] and in detecting unilateral lung re-expansion during bronchoscopy [13].

## 3. The Use of EIT in Neuromuscular Disease Patients: Current Experience and Limitations of Evidence

Most studies that have actually investigated EIT in NMD patients support the use of this technique as a tool for improving the diagnostic pathway for ventilatory compromise. Indeed, EIT was found to identify areas of a non-functioning lung in patients with non-bulbar Amyotrophic Lateral Sclerosis (ALS) by detecting persistent zones of atelectasis, which contribute to reduced electric conductivity through specific lung regions. Moreover, a strong correlation was shown between Forced Vital Capacity (FVC) values and the Impedance Metric (IM), a relative measure of the impedance changes between maximum inspiration and maximum expiration [14]. A moderate-to-strong association was likewise reported between thoracic EIT values and non-normalized PFT results, including FVC, Slow Vital Capacity (SVC), Maximal Inspiratory Pressure (MIP), and Maximal Expiratory Pressure (MEP) in ALS patients, with qualitative differences in comparison to healthy sex- and age-matched controls. The correlations proved to be stronger for supine compared to upright positions; one possible explanation for this finding is that when an individual is lying supine, the posterior EIT electrodes make better contact with the skin since they are being pressed against the thorax [15]. EIT can also be used to measure dynamic lung volume changes in patients with NMD while they are transitioning from an upright to a supine position; in particular, a significant, progressive loss of lung volume during 7 to 10 min of supine positioning was found in ALS subjects with low FVC (<80% predicted) [16]. Unlike previous results, EIT proved ineffective at detecting progressive decline in pulmonary function at the 4-month follow-up visit in ALS patients who simultaneously underwent thoracic EIT measurements and standard SVC in upright and supine positions, despite strong cross-sectional correlations with SVC values. Hypothetically, increased variability in EIT data could explain the lack of sensitivity to changes [17]. Due to the ability to evaluate abnormal patterns of lung ventilation distribution, EIT could potentially play a crucial role in optimizing chest physiotherapy, in particular airway clearance techniques (ACTs), which are a cornerstone of supportive respiratory care in NMD patients. A pilot study, which was recently conducted to investigate the feasibility of using EIT images to estimate the effectiveness of Mechanical Insufflation–Exsufflation (MI-E) on lung volumes in six Type 1 Spinal Muscular Atrophy pediatric patients, reported finding an increase in lung volumes during tidal breathing after intervention in four of the patients [18]. When Casaulta et al. utilized ETI to evaluate the immediate physiological effects of the insufflation/exsufflation technique on ventilation distribution and lung volumes in a group of eight pediatric subjects with different kinds of NMDs, they found that the maneuvers had a negligible short-term effect on lung volumes, expiratory flows, and ventilation distribution. They also reported that it had no influence on the Global Inhomogeneity Index (GI), a parameter that quantifies the tidal volume distribution within the lung [19]. Finally, some investigators designed a physiological pilot study in which EIT was utilized to evaluate the effects of short High-Frequency Percussive Ventilation (HFPV) cycles on lung aeration and gas exchange in a group of tracheostomized patients undergoing mechanical ventilation (MV), including subjects with NMD. The study’s results showed that when HFPV was superimposed onto MV, it promoted alveolar recruitment, as suggested by improved end-expiratory lung impedance (∆EELI), and oxygenation, particularly in patients with high secretion loads [20].

Despite encouraging results, it should be emphasized that current evidence on EIT use in NMD patients has several limitations that can impact the validity and generalizability of the findings. First, the study populations used were quite small, which is usually the case for clinical studies focusing on rare diseases; second, most studies focused on patients with ALS, providing a basis for future utilization which is limited to this specific disease; and third, despite efforts to standardize EIT belt placement, problems with the belt and electrode contact were reported, limiting data quality and interpretation.

The main features of studies evaluating EIT application in NMD patients are outlined in Table 1.

## 4. Conclusions

In summary, current evidence suggests that EIT may offer important advantages compared to conventional Pulmonary Function Tests utilized in NMD patients. However, there are still unsolved technical problems that need to be addressed to facilitate EIT diffusion in clinical practice, in particular sensitivity to electrode–skin interface errors and challenges in reconstruction, which can make accurate imaging difficult. Other persisting limitations are the complexity of applying the technique to obese or kyphoscoliotic patients, and the difficulty of integrating it into soft or flexible wearable devices. Moreover, knowledge about this technique is still not well-developed among physicians caring for NMD patients. Once technical difficulties have been addressed, extensive use of EIT will presumably facilitate the early diagnosis of respiratory compromise, the identification of the disease’s functional phenotype, and proper access to personalized therapies, including new molecules for Neuromuscular Disease. Future research is required to confirm the role of EIT in the clinical care of patients with neuromuscular disorders.

## Figures and Tables

**Table 1:** Evidence on the use of Electrical Impedance Tomography in Neuromuscular Disease Patients (ALS = Amyotrophic Lateral Sclerosis; HFPV = High-Frequency Percussive Ventilation; MI-E = Mechanical Insufflation–Exsufflation; NMD = Neuromuscular Disease; NA = Not Available; PFT = Pulmonary Function Test; SMA = Spinal Muscular Atrophy).

Authors	Type of Study	Population	EIT Analysis	EIT Configuration
Munir B et al. [14]	Physiological pilot study	7 ALS pts 10 healthy volunteers	Correlation with standard PFT	32 channels
Rutkove SB et al. [15]	Physiological study	32 ALS pts 32 healthy controls	Correlation with standard PFT	32 channels
Hansen G et al. [16]	Prospective cohort study	21 ALS pts	Impact of supine posture on lung volumes	16 channels
Rutkove SB et al. [17]	Prospective cohort study	32 ALS pts 32 healthy controls	4 mo decline of pulmonary function	32 channels
Pigatto AV et al. [18]	Physiological pilot study	6 type 1 SMA pts	Effect of MI-E on lung volumes	16 channels
Casaulta C et al. [19]	Physiological pilot study	8 non-ambulatory NMD pts	Effect of MI-E on ventilation distribution	NA
Garofalo E et al. [20]	Physiological pilot study	15 tracheostomized pts	Benefit of HFPV on secretion clearance	16 channels

## Data Availability

No new data were created or analyzed in this study.

## Supplementary Materials

The following supporting information can be downloaded at: https://www.mdpi.com/article/10.3390/jcm14238486/s1, Electric Impedance Tomography: technical notes [21,22,23].
