# Supplementary material for: Rethinking Pulmonary Function Tests in Patients with Neuromuscular Disease: The Potential Role of Electrical Impedance Tomography
Source: J Clin Med. 2025 Nov 29;14(23):8486. doi: 10.3390/jcm14238486 (PMC12692841; doi:10.3390/jcm14238486)
Supplement: Supplementary file 1 [file jcm-14-08486-s001.zip › jcm-3971812-supplementary.pdf]

Electric Impedance Tomography: technical notes

Electric Impedance Tomography (EIT) is able to determine the bioimpedance distribution by injecting low-intensity alternating electrical currents through surface electrodes and measuring the potential differences between pairs of passive electrodes. In accordance with Ohm’s law, the bioelectrical impedance between injecting and measuring electrode pairs is calculated on the basis of the known alternating current and the resulting voltage. Each complete round of impedance measurement of an analyzed section is called a cycle. This process is repeated constantly around the entire thorax during a full cycle, and the impedance changes measured are integrated in a cross-sectional image showing the amount of air in the lung region analyzed.

Current electrical impedance tomographs typically complete 25-50 cycles per second, which translates into 25 to 50 images or frames per second and is consistent with high temporal resolution. The spatial resolution of EIT is instead low, since the cross section of the belt represents an area of approximately 10 cm of the lung in the caudal-cranial direction [1].

EIT images can be divided into regions of interest (ROIs); the waveform over time displays the short- and long-term changes in the local electrical impedance within that region. ROI-based analysis of EIT data provides a more accurate characterization of the spatial heterogeneity of lung ventilation. The information generated by EIT can be analyzed online and offline to detect lung recruitment, derecruitment, overdistension or variations of poorly ventilated lung units [2,3].

References

1. Piraino T. An introduction to the clinical application and interpretation of electrical impedance tomography. *Respir Care.* 2022;67(6):721-9. doi: 10.4187/respcare.09949.
2. Tomicic V, Cornejo R. Lung monitoring with electrical impedance tomography: technical considerations and clinical applications. *J Thorac Dis*. 2019;11(7):3122-35. doi: 10.21037/jtd.2019.06.27.
3. Maciejewski D, Putowski Z, Czok M, et al. Electrical impedance tomography as a tool for monitoring mechanical ventilation. An introduction to the technique. *Adv Med Sci.* 2022;66(2):388-95. doi:10.1016/j. advms.2021.07.010.
